# Acute coronary syndromes: mechanisms, challenges, and new opportunities

**DOI:** 10.1093/eurheartj/ehaf289

**Published:** 2025-05-13

**Authors:** Simon Kraler, Christian Mueller, Peter Libby, Deepak L Bhatt

**Affiliations:** Center for Molecular Cardiology, University of Zurich, Schlieren, Switzerland; Department of Cardiology and Internal Medicine, Cantonal Hospital Baden, Baden, Switzerland; Department of Cardiology and Cardiovascular Research Institute Basel, University Hospital Basel, Basel, Switzerland; Department of Clinical Research, University of Basel, Basel, Switzerland; Division of Cardiovascular Medicine, Department of Medicine, Brigham and Women’s Hospital, Harvard Medical School, Boston, MA, USA; Mount Sinai Fuster Heart Hospital, Icahn School of Medicine at Mount Sinai, 1 Gustave Levy Place, Box 1030, New York, NY 10029, USA

**Keywords:** Acute coronary syndromes, Acute myocardial infarction, Percutaneous coronary intervention, Plaque rupture, Plaque erosion, Plaque healing, Optical coherence tomography, Intravascular ultrasound, Dual antiplatelet therapy, Sex differences, Inflammation, C-reactive protein, Clinical decision-making, Individualized management, Precision medicine, Artificial intelligence, Machine learning

## Abstract

Despite advances in research and patient management, atherosclerosis and its dreaded acute and chronic sequelae continue to account for one out of three deaths globally. The vast majority of acute coronary syndromes (ACS) arise from either plaque rupture or erosion, but other mechanisms, including calcific nodules, embolism, spontaneous coronary artery dissection, coronary spasm, and microvascular dysfunction, can also cause ACS. This ACS heterogeneity necessitates a paradigm shift in its management that extends beyond the binary interpretation of electrocardiographic and biomarker data. Indeed, given the evolution in the global risk factor profile, the increasing importance of previously underappreciated mechanisms, the evolving appreciation of sex-specific disease characteristics, and the advent of rapidly evolving technologies, a precision medicine approach is warranted. This review provides an update of the mechanisms of ACS, delineates the role of previously underappreciated contributors, discusses sex-specific differences, and explores novel tools for contemporary and personalized management of patients with ACS. Beyond mechanistic insights, it examines evolving imaging techniques, biomarkers, and regression- and machine learning-based approaches for the diagnosis (e.g. CoDE-ACS, MI^3^) and prognosis (e.g. PRAISE, GRACE, SEX-SHOCK scores) of ACS, along with their implications for future ACS management. A more individualized approach to patients with ACS is advocated, emphasizing the need for innovative studies on emerging technologies, including artificial intelligence, which may collectively facilitate clinical decision-making within a more mechanistic framework, thereby personalizing patient care and potentially improving long-term outcomes.

## Graphical Abstract

**Graphical Abstract:**
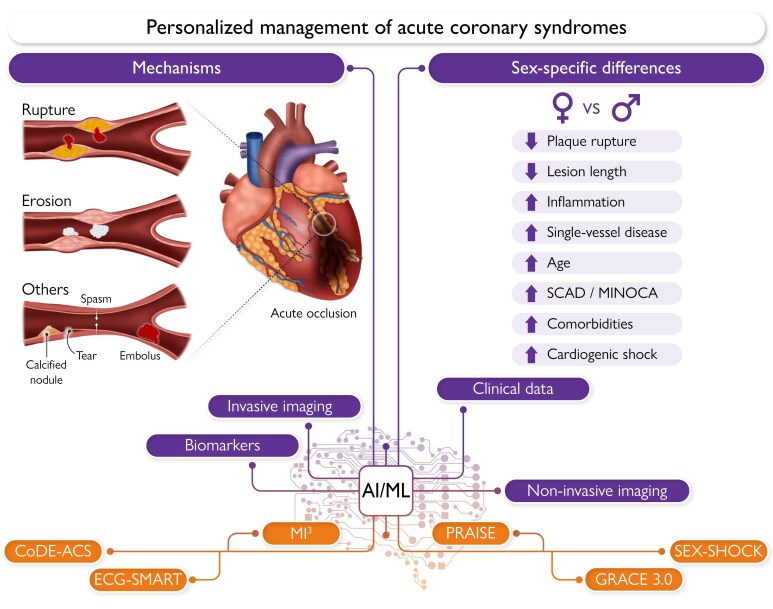

## Introduction

Atherosclerosis and its acute and chronic complications continue to cause most deaths globally.^1,2^ The incidence of acute coronary syndromes (ACS) increases sharply with age in both women and men, though delayed in the former, with underlying mechanisms markedly differing depending on sex, age, and individual risk factor profiles.^2–9^ Most ST-segment elevation myocardial infarction (STEMI) and non-ST-segment elevation (NSTE)-ACS result from an acute reduction in myocardial blood supply due to coronary plaque rupture or erosion, with rupture dominating in STEMI.^10–12^ Other less frequent causes include calcified nodules (CN), spontaneous coronary artery dissection (SCAD), coronary spasm, microvascular dysfunction, and coronary embolism, with the shift in global risk factor profiles contributing to the rise of previously underappreciated mechanisms, including the eroded plaque.^13^ Rupture or fissure usually complicates lipid-rich plaques. Erosion often occurs on proteoglycan-/glycosaminoglycan-rich lesions. Yet, such plaque disruptions may not cause symptoms or provoke an ACS. Such subclinical events can, however, lead to local healing and evolution of plaques.^14^ The pathophysiology of ACS can differ between the sexes, with women more frequently showing non-obstructive disease, less total plaque burden, more often single-vessel disease, and less often plaque rupture as the mechanism triggering ACS, potentially contributing to sex-specific differences in clinical presentation (*Table 1*).^15–28^

**Table 1:** Sex differences in the mechanisms, baseline as well as management characteristics, and outcomes of acute coronary syndromes

		Females	Males	Further details	References
**MECHANISMS/PLAQUE CHARACTERISTICS**	Plaque rupture	↓	↑	Causes up to two-thirds of all ACS STEMI > NSTE-ACS	^5,11,12,15^
	Plaque erosion	(↑)	(↓)	Tends to occur more frequently in females in autopsy studies; however, sex-specific data across intracoronary imaging studies are inconclusive NSTE-ACS > STEMI Increasing relevance due to epidemiological changes More common in premature ACS	^5–8^
	SCAD	↑	↓	Most frequent cause of ACS during pregnancy Territorial preference of the LAD	^16–19^
	MINOCA	↑	↓	Approximately five times more common in females compared with males	^20^
	Number/vessels of non-culprit lesions	↓	↑		^15,21^
	Number of culprit lesions	↔	↔		^15^
	Plaque burden per lesion	↔	↔		^15^
	Lesion length	↓	↑		^15^
	Necrotic core volume	↓	↑		^15,21^
	Calcium content	↓	↑		^8,15,21^
**BASELINE**	Chest pain as the presenting symptom^a^	↓	↑	♂>♀ Chest pain (OR 0.70; 95% CI, 0.63–0.78) Diaphoresis (OR 0.84; 95% CI, 0.76–0.94) ♀>♂ Pain between the shoulder blades (OR 2.15; 95% CI, 1.95–2.37) Nausea or vomiting (OR 1.64; 95% CI, 1.48–1.82) Shortness of breath (OR 1.34; 95% CI, 1.21–1.48)	^22^
	Age during first ACS	↑	↓	Females are ∼7–8 years older at the index ACS	^22–24^
	Pre-hospital delays	↑	↓		^23–25^
	Comorbidity burden	↑	↓	Females are more likely to have diabetes or a history of heart failure	^22–24,26,27^
	Inflammatory burden as gauged by CRP	↑	↓	Might be biased by differences in pre-hospital delay	^24^
	GRACE mortality risk (in-hospital)	↑	↓	Predominantly driven by differences in age	^23,24^
	SCAI Class A	↓	↑	May contribute to the high CS risk among females	^24^
	Single-vessel disease	↑	↓	Similarly observed in patients with premature ACS	^17,24^
**MANAGMENT**	Early invasive (<24 h) strategy	↓	↑	Particularly in NSTE-ACS	^23^
	Coronary angiography	↓	↑		^23,26^
	PCI	↓	↑		^23,26^
	CABG	↓	↑		^23,26^
	Duration of hospital stay at the index ACS	↑	↓		^23,26^
	Guideline-recommended discharge medication	↓	↑	Similarly observed in premature ACS	^17,23,28^
**OUTCOMES**	Incident CS (in-hospital)	↑	↓		^24^
	In-hospital mortality risk (crude)	↑	↓	STEMI > NSTE-ACS	^23,27^
	In-hospital mortality risk (adjusted)	(↔)	(↔)	While published data are inconclusive, effect estimates of crude mortality risk appear to be driven predominantly by clinical and angiographical differences at baseline, particularly in NSTE-ACS	^23,27^
	Benefits of DAPT de-escalation to SAPT (ticagrelor)^b^	(↔)	(↔)	Similar risks of ischaemic but potentially lower major bleeding events (BARC 3 or 5) in both sexes Ticagrelor monotherapy might be linked to a mortality benefit in women, although evidence is currently inconclusive	^29^

ACS, acute coronary syndrome; BARC, Bleeding Academic Research Consortium; CABG, coronary artery bypass grafting; CI, confidence interval; CS, cardiogenic shock; DAPT, dual antiplatelet therapy; DES, drug-eluting stents; GRACE, Global Registry of Acute Coronary Events; LVEF, left ventricular ejection fraction; MI, myocardial infarction; MINOCA, myocardial infarction without obstructive coronary arteries; NSTE-ACS, non-ST-segment acute coronary syndrome; OCT, optical coherence tomography; OMI, occlusion myocardial infarction; OR, odds ratio; PCI, percutaneous coronary intervention; SAPT, single antiplatelet therapy; SCAI, Society for Cardiovascular Angiography and Interventions; SCAD, spontaneous coronary artery dissection; STEMI, ST-segment elevation myocardial infarction. ↑ indicates that the feature is more common, while ↓ indicates the opposite. ↔ indicates no significant sex difference. Parentheses around a symbol signify a trend with inconclusive or limited evidence.

^a^Note, however, that the performance of sex-specific chest pain characteristics for early MI diagnosis is very low, with likelihood ratios close to 1.^232^

^b^As compared to 12 months of DAPT.

The electrocardiogram (ECG) was introduced by Willem Einthoven in 1901,^30^ but ACS management remains to be guided primarily by the presence or absence of ST-segment elevation across contemporary guidelines.^31–33^ The clinical introduction of high-sensitivity cardiac troponin (hs-cTn) assays and the advent of broadly validated assay-specific use-optimized *rule-out* and *rule-in* criteria markedly improved the diagnostic accuracy for acute myocardial infarction (MI), reduced time to decision, and increased the proportion of patients who can be discharged safely from the emergency department.^34^ However, we may need to move beyond this probably overly simplistic ECG and biomarker-based approach to improve the management and outcomes across the wide spectrum of ACS.

For instance, in contrast to MI, unstable angina (UA) may occur in the absence of cardiomyocyte injury, as defined by hs-cTn. Notably, the implementation of hs-cTn assays yielded a ∼20% increase in MI diagnosis in patients with suspected ACS, in part due to reclassification of UA to NSTEMI.^35,36^ Thus, the diagnosis of UA is on the decline.^37^ Patients with UA derive less benefit from an early invasive strategy and intensified antiplatelet strategies and have lower mortality risk than those with confirmed MI,^38^ prompting distinct management strategies.^37^ On the other hand, up to one-fourth of NSTEMI patients have a totally occluded culprit artery at angiography [putting them at 1.7-fold and 1.4-fold increased risk of mortality or major adverse cardiovascular events (MACE), respectively]^39^ and may benefit from an early revascularization strategy.

Despite the success story of ACS care,^40^ contemporary ACS management relies heavily on the binary interpretation of ECG and biomarker data, not reflecting advances in understanding the underlying pathobiology. Through the multidimensional interpretation of clinical, electrocardiographic, imaging, and biochemical data, the use of artificial intelligence and machine learning (AI/ML) will doubtlessly sharpen ACS diagnosis and its prognostication, ushering in a new era of ACS patient management.

## The mechanistic basis of acute coronary syndromes

Plaque rupture continues to lead as a cause of ACS.^10^ Nonetheless, fostered by improved traditional risk factor control, such as aggressive LDL cholesterol (LDL-C) lowering, previously underappreciated mechanisms, including plaque erosion, now assume greater clinical importance.^3^ Changing risk factor profiles, such as increased use of drugs including cocaine,^41^ and nearly unchanged mortality rates in specific ACS patient subgroups necessitate a comprehensive understanding of the diverse mechanisms of ACS to strive for a more personalized approach to the future patient with ACS.

### Plaque rupture: from inflammatory to non-inflammatory triggers

**Figure 1:**
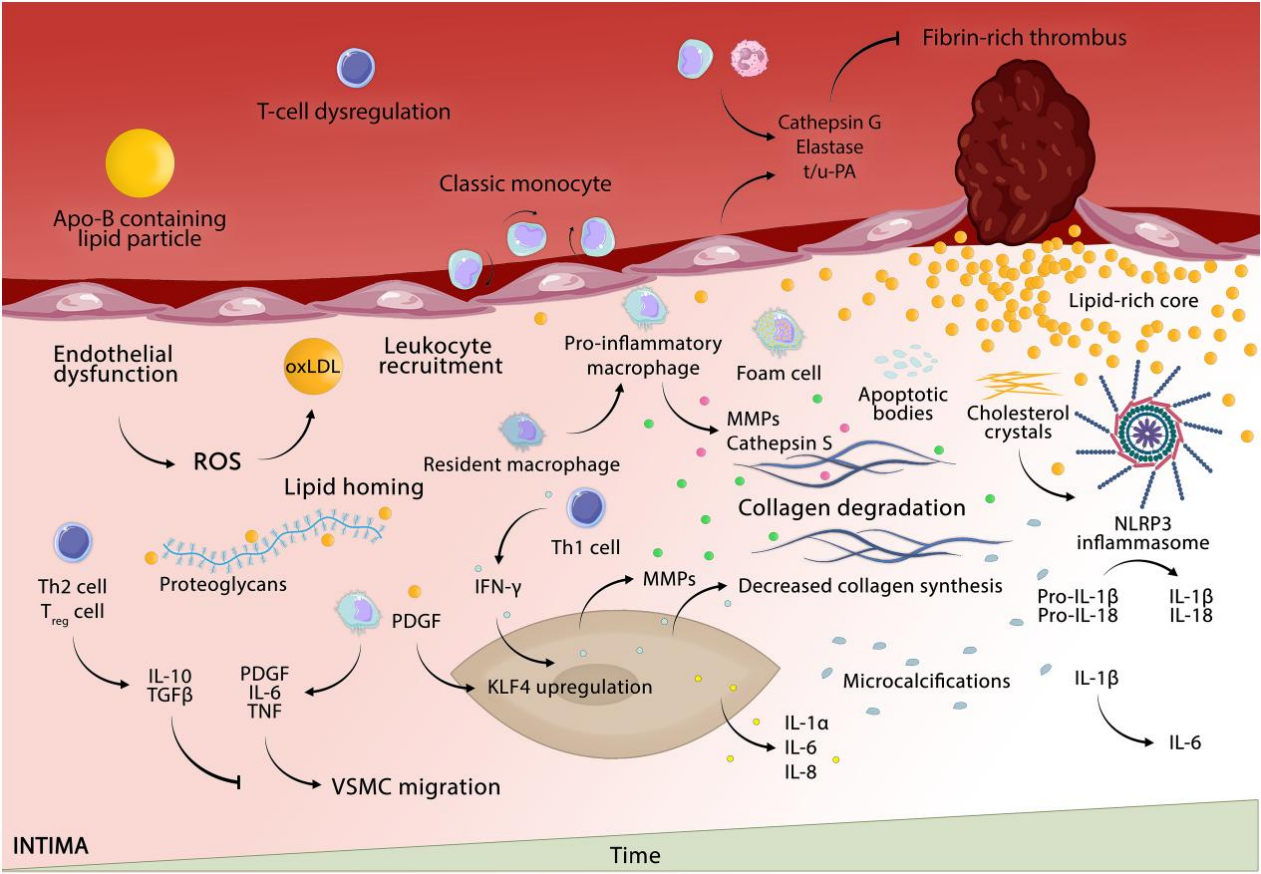
Molecular and cellular mechanisms of plaque rupture—the most common cause of acute coronary syndromes. Plaque formation in the trilaminar coronary artery begins with the subendothelial accumulation of apolipoprotein-B-containing lipid particles [e.g. LDL, lipoprotein(a)]. These lipid particles undergo oxidative and other modifications, triggering a pro-inflammatory response (with subsequent adhesion molecule upregulation) and monocyte recruitment. Classic monocytes migrate into the intima, differentiate into pro-inflammatory macrophages, and internalize modified LDL via scavenger receptors, forming foam cells. The pro-inflammatory milieu and impaired efferocytosis inhibit the polarization of macrophages towards a pro-inflammatory slant. Foam cells then release apoptotic bodies (containing tissue factor amplifying local coagulation), contributing to the accumulation of cholesterol crystals which drives the NOD-like receptor protein 3 inflammasome-mediated interleukin-1β and interleukin-18 activation. Under the influence of growth factors (e.g. platelet-derived growth factor released by activated macrophages and platelets) and certain cytokines, such as interleukin-6, smooth muscle cells then migrate towards the intima and undergo a Krüppel-like factor 4-driven phenotypic modulation. Together with T-helper cell-derived mediators, such as interferon gamma, this results in reduced collagen fibre synthesis and enhanced cathepsin-S and matrix metalloproteinase-mediated collagen breakdown. Imbalances in these pathways, combined with the progressive weakening of the fibrous cap, underpin plaque rupture, the principal driver of acute coronary syndromes. Apo-B, apolipoprotein-B; IFN-γ, interferon gamma; IL, interleukin; KLF4, Krüppel-like factor 4; Lp(a), lipoprotein(a); MMP, matrix metalloproteinase; NLRP3, NOD-like receptor protein 3; oxLDL, oxidized LDL; PDGF, platelet-derived growth factor; ROS, reactive oxygen species; Th, T-helper cell; TNF, tumour necrosis factor; T_reg_, regulatory T-cell; t/u-PA, tissue- or urokinase-type plasminogen activator

The life cycle of an atherosclerotic plaque starts during the second decade of human life, almost half a century before many ACS manifest, with lipid- and foam-cell-rich plaques overlain by a thin fibrous cap (i.e. thin-cap fibroatheroma) considered the most susceptible to rupture. Across contemporary optical coherence tomography (OCT)-based studies, plaque rupture accounted for some 60% of all ACS.^10^ While not all ruptured plaques cause ACS (see section *Mechanisms that contain thrombus formation—‘plaque healing’*), both inflammatory and non-inflammatory mechanisms can perturb the finely regulated structural homeostasis of the fibrous cap triggering plaque disruption (*Figure 1*).

The thinning of fibrous caps accompanies the gradual loss of smooth muscle cells (SMC) and the accumulation of macrophages,^42^ with macrophages characterized by a pro-inflammatory slant dominating the rupture-prone shoulder sites.^43^ The imbalance between extracellular matrix (ECM)-degrading enzymes, such as matrix metalloproteinases (MMPs) and cathepsins, and their corresponding inhibitors, favour rupture of coronary plaques.^44^ Apoptotic lipid-laden macrophages (and erythrocyte-derived membranes) release free cholesterol,^45^ with perturbances of the esterification/de-esterification pathway shifting the free cholesterol-to-esterified cholesterol ratio and facilitating the accumulation of cholesterol crystals (CC).^46^ Notably, CC tend to co-occur with features of complicated plaques and their presence, as detected by OCT, marks an increased risk of MACE after the index ACS.^47^ While multifaceted mechanisms may contribute to this phenomenon, CC can co-stimulate the NOD-like receptor protein 3 (NLRP3) inflammasome that activates the precursors of interleukin (IL)-1β and IL-18.^48^

Respiratory infections, such as influenza (especially influenza B), can also precipitate ACS,^49–51^ though the mechanisms are less well explored, likely involving a T-cell-mediated (auto-)immune response coupled with a prothrombotic state in genetically susceptible individuals (reviewed by Epstein *et al*.^52^ and Libby *et al*.^53^).^54^ Adaptive immunity also plays an important role in maintaining plaque stability, with ACS patients having notably dysregulated CD4^+^ T-cell subpopulations (reviewed by Flego *et al*.^55^), including a higher frequency of T-helper cell 1 (Th_1_) [the most prominent CD4^+^ T-cell population infiltrating atherosclerotic plaques and an important source of interferon gamma (IFN-γ)], CD28^null^ (a source of IFN-γ and TNF), and Th_17_ cells (a source of IL-17), while having attenuated regulatory T cells (T_reg_), both quantitatively and qualitatively, that compromise their physiological ability to maintain immune homeostasis.

Non-inflammatory triggers, including extreme emotional or physical stress, may also provoke plaque rupture. Upon extreme physical exertion,^56^ sexual activity,^57^ terror attacks, or environmental catastrophes,^58,59^ the *fight or flight* response activates the sympathetic nervous system and release of adrenal catecholamines, with their β_1_-receptor-mediated positive chrono-/inotropic effects and α_1_-receptor-mediated vasoconstriction causing blood pressure levels to raise, and through their effects on platelet aggregation,^60^ inducing a prothrombotic state. Pioneering cell-tracking experiments have further shown that acute mental stress facilitates leukocyte recruitment into plaques through increased adhesion molecule expression and chemokine release, mainly involving locally derived norepinephrine.^61^ These responses can contribute to the triggering of plaque disruption and thus provoke ACS. In support of this notion, admission diagnoses of MI transiently increased from 11.2% to 15.5% at a hospital adjacent to the World Trade Center in the 2 months following 9/11, while diagnoses of UA declined.^59^ The sympathetic nervous system also drives the high ischaemic risk that persists following an ischaemic event, which relates, at least in part, to the accelerated β_3_-receptor-mediated increased myelopoiesis and monocyte recruitment,^62,63^ as well as its effects on trained immunity that can programme long-term ischaemic risk.^64^

### The eroded plaque in an era of changing risk factor profiles

The estimated prevalence of plaque erosion appears to have increased in recent decades, now causing up to one-third of ACS, with a higher proportion in NSTEMI. The advent and increasing deployment of effective LDL-C-lowering therapies can reduce the lipid content of plaques, reinforcing the collagen content of the fibrous cap, which lessens the risk of rupture but contributes to the relative rise of plaque erosion.^65,66^ Indeed, plaque erosion tends to occur more frequently in those with better controlled LDL-C and blood pressure.^6^ Eroded lesions also tend to localize near bifurcation sites, where a high oscillatory shear index (OSI; i.e. reversing directions of endothelial shear stress [ESS]) prevails.^67^ These conditions can augment the expression of toll-like receptor (TLR)-2, an innate immune signalling molecule implicated in endothelial injury.

**Figure 2:**
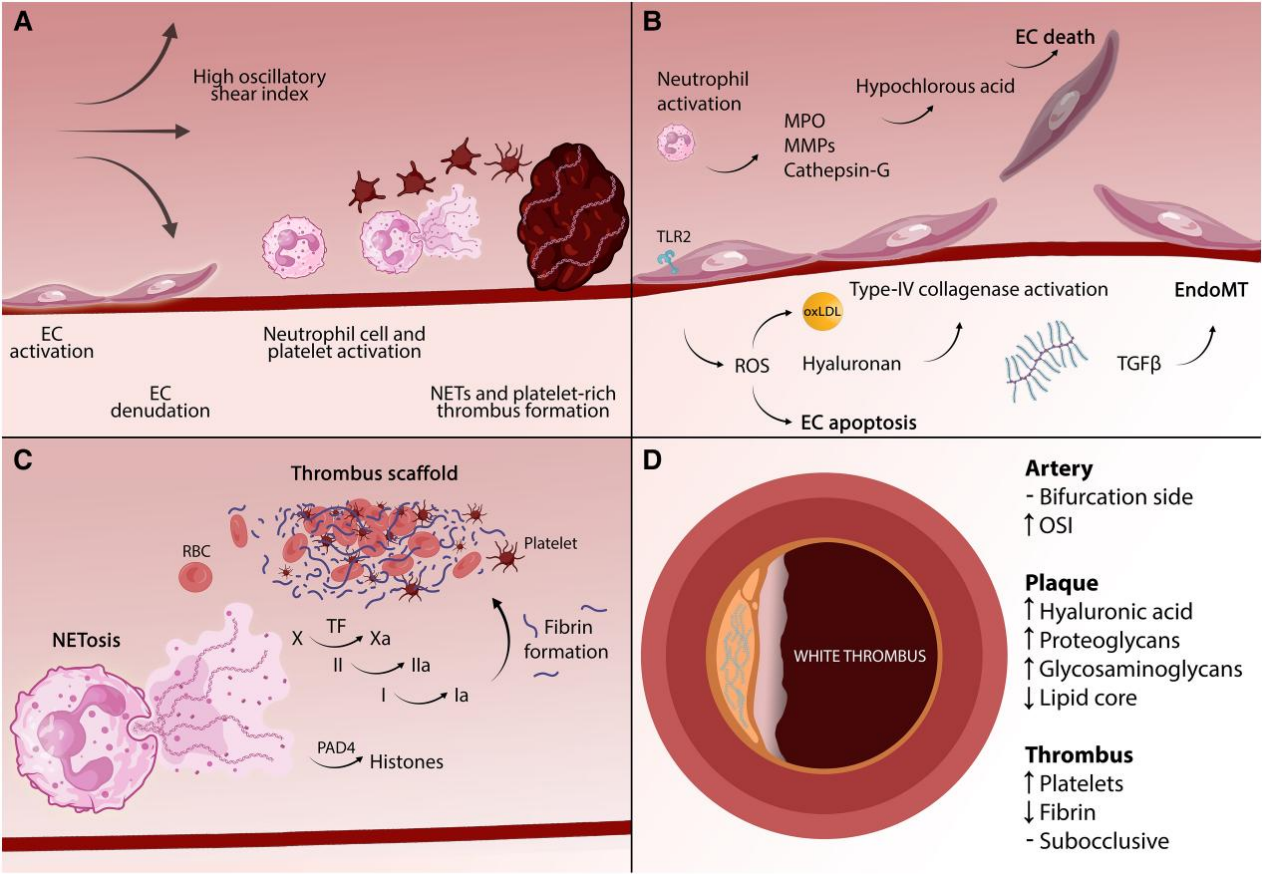
Mechanistic insights into plaque erosion—an increasingly prevalent cause of acute coronary syndromes. (*A*) In regions of high oscillatory shear index (i.e. a measure of the changes in shear stress vectors over the cardiac cycle), EC activation and denudation, followed by neutrophil cell and platelet activation, are followed by NET and platelet-rich thrombus formation, resulting in plaque erosion, the second most common cause of acute coronary syndromes. (*B*) Toll-like receptor 2 ligation (e.g. through hyaluronan fragments) induces EC activation and intracellular reactive oxygen species synthesis, promoting type-IV collagenase activation (e.g. matrix metalloproteinase-2 or 14), endothelial desquamation, and EC apoptosis. While matrix metalloproteinases degrade basement membrane components, neutrophil-derived myeloperoxidase produces hypochlorous acid, thereby exacerbating EC death and TF expression. In parallel, certain stimuli (e.g. transforming growth factor-β) augment the endothelial loss of squamous morphology and their apical-basal polarization, driving endothelial to mesenchymal transition and their intimal penetration. (*C*) The exposure of subendothelial structures to circulating platelets and neutrophils leads to NET formation, with NETs acting as scaffolds for TF and coagulation cascade activation. Neutrophil extracellular trap formation may depend on PAD4, an enzyme that catalyses arginine to citrulline conversion, thereby altering the ionic interactions between DNA and proteins within the histone structure. Finally, activated platelets aggregate via fibrin cross-linking, forming a platelet-rich (‘white’) thrombus. (*D*) Plaque erosion typically occurs in arteries with disturbed flow dynamics, such as bifurcation sides with a high oscillatory shear index. Eroded plaques contain abundant hyaluronic acid, proteoglycans, and glycosaminoglycans, with minimal or no lipid cores. The resultant thrombi are platelet-rich, fibrin-poor, often causing subocclusive thrombosis. EC, endothelial cell; EndoMT, endothelial-to-mesenchymal transition; MMP, matrix metalloproteinase; MPO, myeloperoxidase; NETosis, neutrophil extracellular trap formation; NETs, neutrophil extracellular traps; OSI, oscillatory shear index; oxLDL, oxidized LDL; PAD4, protein arginine deiminase 4; RBC, red blood cell; ROS, reactive oxygen species; TFG-β, transforming growth factor-β; TF, tissue factor; TLR2, Toll-like receptor 2

Endothelial cell (EC) desquamation, followed by local platelet and neutrophil activation, neutrophil extracellular trap (NET) release, and platelet-rich thrombus formation at the site of intimal denudation hallmark plaque erosion (*Figure 2*). During NET formation, granulocytes release unwound DNA along with intracellularly stored reactive oxygen species (ROS), proteinases (e.g. MMP9, cathepsin G), and other prothrombotic/pro-inflammatory proteins including myeloperoxidase (MPO), an enzyme that is elevated in plasma of patients with ACS caused by plaque erosion.^68–70^ The product of MPO, hypochlorous acid, promotes EC death and desquamation.^71^ Disturbed flow and the NLRP3-activated cytokines IL-1β and IL-18 aid the local recruitment of neutrophils,^13,72–74^ which can form NETs. Neutrophil extracellular traps participate in thrombus formation on the eroded plaque (reviewed by Döring *et al*.^75^). Indeed, the meshwork of nuclear/mitochondrial DNA strands furnishes a retiform scaffold and favours thrombus propagation through platelet entrapment.^76,77^ Genetic or pharmacological reduction of NET formation blunts its deleterious effects on endothelial structure and function in hyperlipidaemic mice.^78^ While statins may augment NET formation,^79^ aspirin appears to attenuate this process.^80^ During plaque erosion, platelets superimposed on the denuded endothelium, activated by contact with the now naked collagenous subendothelial ECM, release their granular contents, aggregate, and form a platelet-rich thrombus which may eventually provoke ACS.

Hyaluronic acid, a constituent of the erosion-prone plaque, can signal through *TLR2*.^69,81^  *In vitro*, *TLR2* ligation of cultured human ECs induces their activation and intracellular ROS production, priming them towards cell death and detachment, processes that are aggravated by neutrophils.^73^ Endothelial-to-mesenchymal transition may also accompany erosion and influence plaque healing.^82,83^ In carotid endarterectomy specimens, ECs bearing markers of apoptosis were ∼6.9 more abundant distal to the stenosis,^84^ a region where disturbed ESS predominates and eroded plaques frequently localize. Indeed, in OCT-based work using computational fluid dynamics, most thrombi caused by erosion developed between areas of high ESS and low ESS/high OSI.^85^

### Mechanisms that contain thrombus formation—‘*plaque healing*’

**Figure 3:**
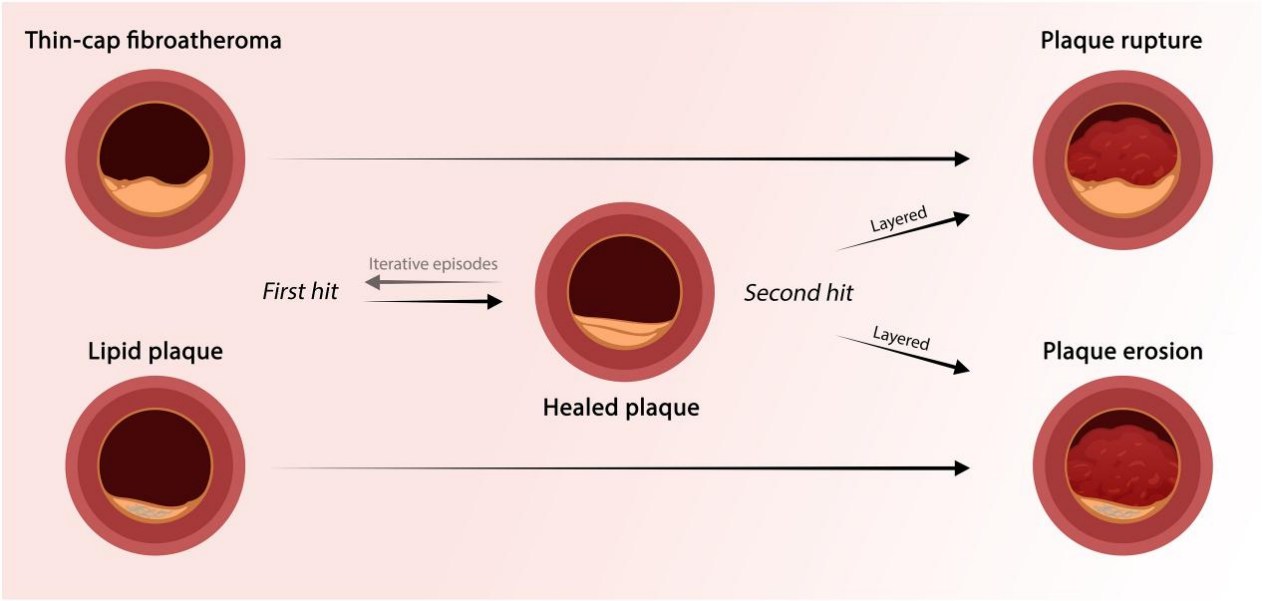
Not all plaques trigger acute coronary syndromes: mechanistic insights into plaque healing. Thin-cap fibroatheromas (top left) with a lipid-rich core and macrophage accumulation represent a high-risk plaque prone to rupture. Conversely, proteoglycan- and glycosaminoglycan-rich (fibrous) plaques (bottom left) may form the basis of plaque erosion. The cellular mechanisms of plaque rupture (top right) or erosion (bottom right) (‘*first hit*’) causing (sub-)occlusive thrombosis in the setting of impaired healing capacity (‘*second hit*’) are shown in *Figs. 1* and *2*, respectively. Healed plaques (centre) are characterized by reparative processes that follow plaque disruption by rupture or erosion. Proliferating SMC may synthesize ECM components, such as proteoglycans and type-III collagen, forming a provisional matrix. Over time, this matrix is replaced by mature type-I collagen, stabilizing the plaque, and promoting its re-endothelialization. Effective healing capacity contains the first hit, stabilizing the plaque and promoting its evolution into a layered, less thrombogenic and disruption-prone lesion. Repeated cycles of subclinical disruption and healing may lead to progressive luminal narrowing and the development of stable but stenotic lesions without acute clinical events. ECM, extracellular matrix; SMC, vascular smooth muscle cells

About one out of three to four patients with ACS have layered (‘*healed*’) plaques at the culprit site,^86,87^ suggesting that many disrupted lesions do not result in an ACS (*Figure 3*).^14,88,89^ Such subclinical disruption events nonetheless provoke healing that can drive their progression towards a fibro-calcific phenotype, which may induce flow-limiting stenoses, but lesions that may have less propensity to rupture.^90^ Plaque healing can involve three overlapping phases: thrombus lysis and/or organization, wound healing, and re-endothelialization (reviewed by Vergallo and Crea^14^). Local thrombolysis may involve tissue plasminogen activator and urokinase plasminogen activator produced by EC and SMC along with leukocyte-derived cathepsin G and elastase. Healing of disrupted plaques involves platelet-derived growth factor (PDGF)-BB and transforming growth factor-β (TGF-β) which stimulate SMC migration and ECM production. Thus, a new layer of matrix forms over the disrupted lesion, yielding the intravascular imager’s layered plaque or the pathologist’s buried fibrous cap. Newly laid down type-III collagen can be joined by type-I collagen, followed by re-endothelialization of the disrupted plaque surface. Such neointima formation can restore arterial integrity. These lesions, reinforced by new ECM, may have less tendency to rupture, but through intimal expansion and constrictive remodelling may evolve into flow-limiting stenoses. The OCT-based study of Vergallo *et al*.^91^ supports this scenario by showing that healed plaques in non-culprit coronary segments occur roughly nine times more often in chronic coronary syndrome patients compared with those who have recurrent ACS, aligning with autopsy data obtained in 39 coronary specimens that strongly link features of plaque healing with degree of luminal stenosis.^89^ Secondary prevention measures, including lipid-lowering diet, statins, PCSK9 inhibitors, and antiplatelet and anti-inflammatory regimens may promote plaque healing (*Table 2*).

**Table 2:** Available and emerging secondary prevention measures and how they may promote plaque healing

Intervention	Drug class	Mechanism of action	Potential favourable effects on plaque healing	References
Lipid-lowering therapies	Statins	Lipid-lowering dependent and independent mechanisms	Macrophage infiltration↓, MMP-(1, 2, 3, 9) expression↓, oxidative stress↓, adhesion molecule expression↓, SMC maturation↑, collagen synthesis↑, TF expression↓, PAI-1 expression↓, cholesterol crystallization↓	^92,93^
	PCSK9 inhibitors		Collagen content↑, MMP-9 expression↓	^94^
	Cholesterol absorption inhibitors		Macrophage differentiation↓, oxidative stress↓, cholesterol crystallization↓	^95–97^
	ATP citrate lyase inhibitors		Collagen content↑, apoptosis↑, efferocytosis↑	^98^
	*AnGPTL3 inhibitors?*		Macrophage infiltration↓	^99^
	*CETP inhibitors?*		Collagen↑, macrophage infiltration↓, SMC infiltration↑	^100^
Lipid-lowering diet	N/A		Macrophages↓, collagen content↑, oxidative stress↓, TF expression↓	^101–103^
Antiplatelet therapy	Aspirin	Irreversible cyclooxygenase inhibition	Macrophage infiltration↓, SMC infiltration↑, collagen content↑, cholesterol crystallization↓	^104,105^
Anti-inflammatory therapy	Colchicine	Predominant suppression of leukocyte trafficking (through the inhibition of actin polymerization) and, at higher doses, likely also of the NLRP3 inflammasome	Macrophage function↓, NET production↓, collagen content↑	^106–108^
	*IL-6 inhibition?*	t.b.d.	Macrophage infiltration↓, MMP-9 expression, SMC infiltration↑, collagen↑	^109,110^
	*Icosapent ethyl*	Induces an anti-inflammatory transcriptome in cultured CD4^+^ T cells	Macrophage infiltration↓, MMP-(2,9) expression, adhesion molecule expression↓, SMC infiltration↑, collagen content↑, oxidative stress↓, T-cell modulation	^111–114^

AnGPTL3, angiopoietin-like protein 3; ATP, adenosine triphosphate; CETP, cholesteryl ester transfer protein; MMP, matrix metalloproteinase; N/A, not applicable; NET, neutrophil extracellular trap; NF-κB, nuclear factor kappa B; NLRP3, NOD-, LRR-, and pyrin domain-containing protein 3; PAI-1, plasminogen activator inhibitor-1; PCSK9, proprotein convertase subtilisin/kexin Type 9; PPAR-α, peroxisome proliferator-activated receptor alpha; t.b.d., to be determined; TF, tissue factor; SMC vascular smooth muscle cells. ↑ signifies an increase, while ↓ indicates a decrease.

### Looking beyond the ruptured or eroded plaque: other mechanisms of acute coronary syndromes

While plaque rupture or erosion trigger the vast majority of ACS, CN, SCAD, coronary spasm, embolism, and microvascular dysfunction can also cause ACS.^9^ Indeed, CN may become increasingly relevant as the population ages.^115^ Nonetheless, insights into the underlying molecular and cellular mechanisms of ACS beyond plaque rupture or erosion remain very limited.

According to the current concept, protruding CN, predominantly described in older patients with equal prevalence in women and men, may disrupt the overlying fibrous cap and, through the loss of EC, can cause (typically subocclusive) thrombosis and subsequent ACS.^116^ The precise mechanisms of CN formation remain largely unexplored, but as CN may occur more frequently in patients with chronic kidney disease,^115,117,118^ perturbed calcium phosphate homeostasis may contribute to its pathogenesis.^119^ Furthermore, CN are associated with features of plaque healing,^116^ and preferentially affect the mid- to proximal segments of the right coronary artery, a region of maximal hinge motion during each cardiac cycle.^115,116,118^

The umbrella term MI with non-obstructive coronary arteries (MINOCA) refers to ACS in the absence of haemodynamically significant coronary stenoses (<50% of any major epicardial vessel during coronary angiography). Myocardial infarction with non-obstructive coronary arteries typically occurs in the absence of traditional risk factors and preferentially affects younger patients (second most common cause of premature ACS, accounting for 10%–20% of cases in the young)^41^ with a clear female predominance (approximately five-fold increased odds compared with males).^20^ While MINOCA can occur on the basis of plaque disruption (plaque rupture, erosion, or CN), leading to distal embolization, spasm, or transient occlusion, the vast majority appear to arise from non-atherosclerotic causes, including SCAD, coronary spasm (with a high prevalence among East Asians, also frequently linked to recreational drug use),^120^ embolism (with an association with atrial fibrillation in STEMI patients),^121^ and microvascular dysfunction (reviewed by Agewall *et al*.^122^). Cocaine, marijuana, (meth)amphetamine, sumatriptan, inotropic agents, extreme stress, hypertension, and smoking can precipitate MINOCA.^20^ Hence, eliciting a history and testing for recreational drug use should be considered, especially among those with premature ACS. The causal role of inherited or acquired thrombophilia in MINOCA pathogenesis remains highly controversial, but some data suggest an increased frequency of Factor V Leiden and the prothrombin variant G20210A in these patients,^123^ with the pooled prevalence of thrombophilia being 14% across eight studies.^120^ The spectrum of MINOCA aetiologies and relevant differential diagnoses (e.g. myocarditis, Takotsubo cardiomyopathy) are broad, making functional coronary angiography and/or multimodality imaging, including cardiac magnetic resonance (CMR), a necessity.^124^

Spontaneous coronary artery dissection, a sudden tear in the coronary artery wall, also tends to affect primarily young ACS patients, particularly women.^17^ Spontaneous coronary artery dissection occurs predominantly in the absence of traditional risk factors,^19^ affects 7%–35% of female ACS patients ≤50 years,^17,18^ is the most frequent cause of ACS in the peripartum,^16^ and often affects the left anterior descending coronary artery.^19^ Our mechanistic understanding is incomplete (reviewed by Hayes *et al*.^125^), but SCAD is initiated by an intimal tear or medial disruption, resulting in the formation of an intramural haematoma, thereby compressing the true lumen, impeding myocardial blood supply, and potentially causing ACS. Its aetiology is multifaceted, with (inherited) arteriopathies (e.g. fibromuscular dysplasia), connective tissue disorders (*FBN1*-mutation caused Marfan, *COL3A1*-mutation caused Ehlers–Danlos syndrome, and Loeys–Dietz syndrome, the latter caused by mutations in TGF-β-dependent signalling cascades), hormonal changes (e.g. peripartum period with a clustering in the first week after delivery, hormone therapy), systemic inflammatory conditions (e.g. SLE, sarcoidosis, chronic inflammatory bowel diseases), and genetics contributing importantly to its pathogenesis,^125^ with ≥50% of SCAD patients reporting extreme emotional (female predominance) or physical (male predominance) stressors precipitating the ACS.^126^

## Premature acute coronary syndromes: the need for refined risk factor concepts

Historically, ACS typically affected middle-aged, hypercholesterolaemic male smokers with hypertension.^127^ However, population ageing coupled with emerging risk factors which young individuals now increasingly encounter (e.g. vaping, oral smokeless tobacco products, marijuana, cocaine) has changed ACS epidemiology: the total numbers of admissions for ACS declined in the USA,^128^ while incidence among young patients remained stable.^65^ Some one in four patients presenting with ACS are <55 years old.^129,130^ Among these patients, those without standard modifiable cardiovascular risk factors (SMuRFs; potentially representing patients with underdiagnosed, incompletely controlled, or *yet-to-be-identified* risk factors) deserve particular focus. Indeed, these patients display lower prescription rates for guideline-directed medical therapy and in some studies are at higher mortality risk,^131,132^ and may benefit from an intensive preventive approach, as proposed by Figtree *et al*.^133^.

**Figure 4:**
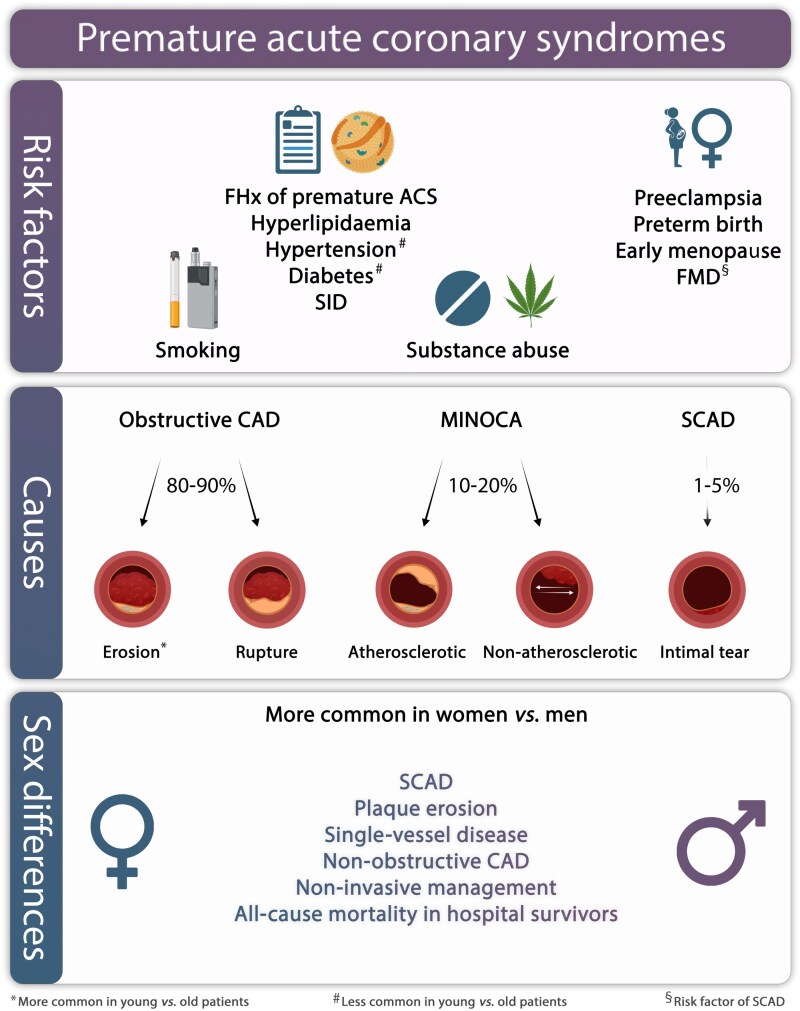
Risk factors (top), causes (centre), and sex differences (bottom) of premature acute coronary syndromes. Risk factors for premature acute coronary syndromes include smoking (tobacco, marijuana, or vaping), a family history of premature acute coronary syndromes, dyslipidaemias [high lipoprotein(a), LDL cholesterol], systemic inflammatory diseases, hypertension, and diabetes, though the latter two are intriguingly less frequently observed in young vs old patients presenting with acute coronary syndromes. Substance abuse (particularly sympathomimetic drugs such as cocaine) remain a significant risk factor for premature acute coronary syndromes, with female sex, pregnancy, and fibromuscular dysplasia enhancing the susceptibility for spontaneous coronary artery dissection (affecting 7%–35% of female acute coronary syndrome patients ≤50 years and representing the most frequent cause of acute coronary syndrome during pregnancy). Most common causes of premature acute coronary syndrome include obstructive coronary artery disease (80%–90%), myocardial infarction with non-obstructive coronary arteries (10%–20%), and spontaneous coronary artery dissection. Plaque erosion, non-obstructive coronary artery disease, spontaneous coronary artery dissection, single-vessel disease, and congenital coronary anomalies dominate in women. Young women who survived hospitalization due to acute coronary syndromes have higher all-cause mortality risk as compared to young men. CAD, coronary artery disease; FHx, family history; FMD, fibromuscular dysplasia; MACE, major adverse cardiovascular events; MINOCA, myocardial infarction with non-obstructive coronary arteries; SCAD, spontaneous coronary artery dissection; SID, systemic inflammatory disease

Patients with premature ACS have mechanisms similar to those in older counterparts, but their prevalence differs markedly (reviewed by Rallidis *et al*.^41^). While causes may overlap or even coexist, we categorize them into three major groups for conceptual clarity: (i) ACS with (sub-)occlusive coronary artery disease, (ii) MINOCA, or (iii) SCAD, as discussed above. Obstructive coronary atherosclerosis, as in older patients, accounts for the majority of ACS in young patients (∼80%–90%) (*Figure 4*).^41^ Likely driven by the shorter cumulative exposure to traditional risk factors, coronary plaque burden and progression is less extensive (shorter lesion lengths, lower plaque volumes, and more often single-vessel disease), particularly among females, though young women tend to have worse long-term outcomes relative to young males.^17,134–136^ Acute coronary syndrome-causing plaques also differ in their composition: lesions in younger patients typically have more fibrotic character, smaller necrotic cores, but less compensatory luminal enlargement or positive remodelling).^134^ Optical coherence tomography-based studies suggest a lower prevalence of thin-cap fibroatheromas and spotty calcification, possibly contributing to the higher prevalence of plaque erosion in young (particularly frequent among premenopausal women) vs old ACS patients.^136,137^ While risk factors of obstructive ACS largely match those seen in older patients, their prevalence differs considerably: a positive smoking history (tobacco, marijuana, vaping; ∼50%–95%), dyslipidaemias (∼60%–95%), a family history of premature coronary artery disease (∼30%), and cocaine abuse (∼5%) are by far more prevalent in young ACS patients, while they have less diabetes and hypertension.^135,138,139^ The effects of tobacco smoking on plaque progression and instability have been reviewed in detail elsewhere,^140,141^ but the mechanisms by which marijuana and vaping provoke ACS remain largely unexplored, despite the alarming rise in their use, increased potency, and dose-dependent association with cardiovascular risk (similar to and independent of tobacco use).^138,142,143^ Mechanisms through which marijuana triggers ACS are likely multifaceted (reviewed by DeFilippis *et al*.^144^); nonetheless, the markedly increased ischaemic risk within 60 min following its consumption favours the involvement of an acute, likely sympathetic nervous system-mediated response (thereby raising myocardial oxygen demand while increasing the susceptibility to vasospasm).^145^

Similarly, through the presynaptic inhibition of norepinephrine and dopamine reuptake, cocaine triggers a positive chronotropic (aggravated with alcohol consumption^146^) and inotropic response (with a synergistic effect of tobacco smoking^147^), with its α-adrenergic effects on SMC causing coronary vasoconstriction, which may collectively result in ACS.

More than half of patients with premature ACS show evidence of dyslipidaemia, with 10%–20% and 12.5%–38% bearing a probable diagnosis of heterozygous familial hypercholesterolaemia and combined hyperlipidaemia, respectively.^148–151^ In a case–control study, Lp(a) levels >50 mg/dL (present in up to one out of three patients with premature ACS^152^) conferred an approximately three-fold increased risk of premature ACS.^153^ Hence, all patients, especially those with premature ACS, should have a measurement of Lp(a). Systemic inflammatory conditions represent another risk factor for coronary artery events in the young. Indeed, patients with type 1 MI and a history of chronic inflammatory conditions such as rheumatoid arthritis or lupus erythematosus had worsened long-term survival in the *Mass General Brigham YOUNG-MI Registry*.^154^ The study by Hindieh *et al*.^155^ further highlights a genetic predisposition to premature ACS. Indeed, among 763 patients with premature ACS, either a positive family history or a high genetic risk score was independently and additively associated with increased plaque burden and more severe stenosis on angiography. In agreement, a polygenic risk score most strongly predicted event recurrence among patients with premature ACS participating in the *Italian Genetic Study of Early-Onset Myocardial Infarction*.^156^ Whether preventive strategies in such high-risk individuals, as assessed by (poly-)genetic risk scores, reflect into improved patient outcomes remains to be investigated, representing an exciting avenue for future research.

## Tools for personalized care

Epidemiological shifts, changing risk factor profiles, and the intricacy of ACS pathobiology necessitate a precision medicine approach to female and male patients with ACS. Genomics can guide dual antiplatelet therapy (DAPT) de-escalation (e.g. in CYP2C19*2 or CYP2C19*3 loss-of-function alleles carriers) and statin therapy optimization.^31,32,157–159^ However, the utility of these measures to improve outcomes is uncertain and the optimal duration of DAPT remains controversial.^160^ By harnessing novel biomarkers and imaging- and AI/ML-based tools, we should strive for more personalized ACS management concepts that go beyond the STEMI/NSTE-ACS dichotomy.

### Non-invasive cardiac imaging

Computed tomography (CT), frequently used in clinical practice to rule out pulmonary embolism or aortic dissection, has evolved considerably over the last two decades. Coronary CT angiography (CCTA) may well supplant many diagnostic uses of conventional diagnostic coronary angiography.^161^ Indeed, the steadily increasing spatiotemporal resolution of CT scanners and the introduction of photon-counting detectors now allow an in-plane resolution of up to 110 µm,^162^ ushering in a new era of CCTA and the personalized assessment of coronary lesions (reviewed by Nurmohamed *et al*.^163^). Presently, CCTA is not recommended for routine use in patients with ACS, but it provides high negative predictive value for coronary artery disease (if ECG and hs-cTn results are inconclusive) and thus can serve to risk stratify selected patients with NSTE-ACS.^31,32,164–166^ Many ACS culprit lesions are non-obstructive^167^ but exhibit qualitative features associated with risk of disruption. Artificial intelligence and machine learning-assisted analyses of CCTA promise to provide non-invasive quantitative and objective assessment of such coronary plaque characteristics.^163^ In a complementary manner, CMR imaging allows the comprehensive assessment of myocardial structure and function, specifically the visualization of myocardial injury, oedema, fibrosis, scarring, perfusion, and tissue viability. Cardiac magnetic resonance can aid the identification of causes of MINOCA (e.g. myocarditis, takotsubo cardiomyopathy), as well as of intracardiac thrombi. Similarly, transthoracic echocardiography can aid the detection of ongoing ischaemia or previous MI and the investigation of differential diagnoses (e.g. aortic dissection, pulmonary embolism) and remains the cornerstone of the emergency work-up of ACS patients with haemodynamic instability or cardiogenic shock (CS). Going forward, harnessing AI/ML models that merge laboratory biomarker and imaging data may enable the identification of the mechanism of ACS in a given patient non-invasively informing personalized ACS management.^168,169^

### Intracoronary imaging

**Figure 5:**
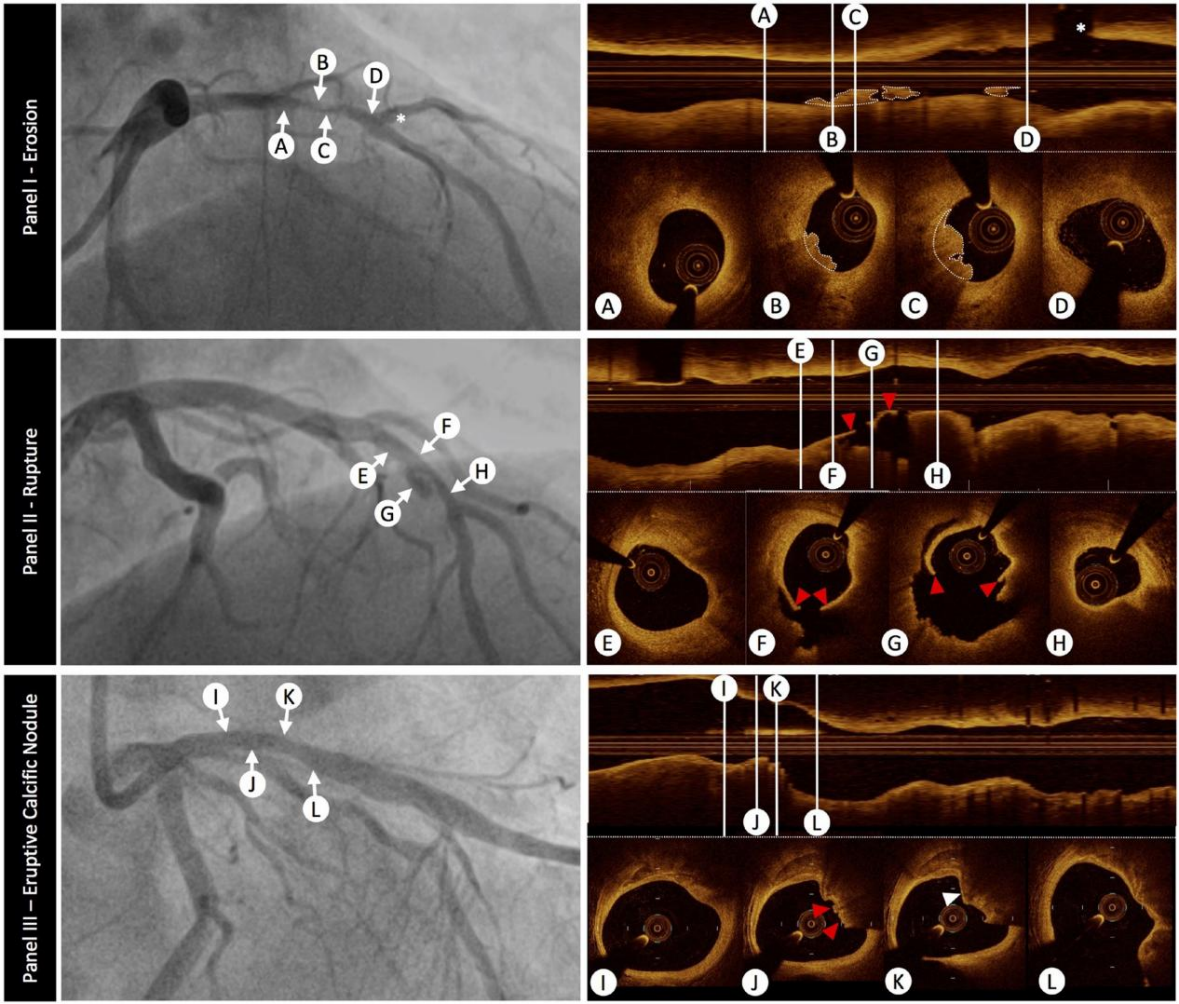
Intracoronary imaging for the detection of plaque rupture, erosion, and calcified nodules. Panel I—plaque erosion: coronary angiography (left) shows severe stenosis in the mid-left anterior descending coronary artery (arrows B, C), where plaque erosion most frequently occurs. Optical coherence tomography (right) reveals an irregularly shaped luminal surface with attached mural thrombus (dotted outline) overlying the fibrous plaque (B, C) in the absence of plaque rupture. Fibroatheromas are visible proximal (A) and distal (D) to the subocclusive thrombus near a diagonal branch (asterisk). Panel II—plaque rupture: the culprit lesion is in the left anterior descending coronary artery (arrows F, G), with optical coherence tomography identifying a disrupted fibrous cap (red arrowheads) and a notable plaque cavity (F, G). Thin-cap fibroatheromas are detected in the proximal (E) and distal (H) segments of the lesion. Panel III—calcified nodules: coronary angiography shows a lesion in the proximal part of the left anterior descending coronary artery (arrows J, K). Optical coherence tomography reveals calcific infiltration of the vessel wall, with a superficial calcific sheet (I, 11–2 o’clock) and disruption of the luminal contour with overlying red thrombus (J, red arrowheads). An irregular calcified nodule (K, white arrowhead) causes significant image attenuation, potentially mimicking a thrombus, with endothelial integrity being preserved distally (L). Reprinted from Johnson *et al*.^171^

High-resolution intracoronary OCT and intravascular ultrasound (IVUS) add information to 2D coronary angiography, allowing the assessment of arterial anatomy, aspects of plaque composition, stent deployment, and PCI results (*Table 3*).^170,171^ Well established as research tools,^172^ clinical use of these modalities offers an opportunity to determine plaque characteristics and thrombus burden, tailoring ACS stenting strategies. Their use can improve long-term outcomes, as evidenced by a recent RCT in ACS patients,^173^ and a network meta-analysis showing a 29% risk reduction of target lesion failure by intracoronary imaging- vs a sole coronary angiography-guided revascularization strategy during PCI.^174^ Because it has ten-fold higher resolution than IVUS (10–15 vs 100–150 µm), OCT can differentiate between plaque rupture (disrupted fibrous cap overlying a lipid-rich core), erosion (intact fibrous cap with superimposed thrombus), CN (disrupted fibrous cap with protruding calcium), and SCAD in many patients (*Figure 5*). Plaque erosion is a diagnosis of exclusion: it is considered ‘*definite*’ if a thrombus overlies a lesion with a demonstrable intact fibrous cap but only ‘*probable*’ if an irregular contour or thrombus is present without manifest fibrous cap integrity.^5^

**Table 3:** Selected invasive and non-invasive imaging modalities beyond intracoronary angiography

	CCTA	IVUS	OCT
Invasive technique	No	Yes	Yes
Suitable for AI/ML-assisted analyses	Yes	Yes	Yes
Whole-heart coronary imaging	↑↑↑	↑	↑
Assessment of			
Plaque volume	↑↑↑	↑↑↑	↑
Plaque composition	↑↑	↑↑	↑↑↑
Presence of macrophage infiltration			↑
Calcium quantification	↑↑	↑	↑↑↑
Lipid core	↑↑	↑	↑↑
TCFA			↑↑↑
Plaque rupture		↑	↑↑↑
Intraluminal thrombus	↑	↑↑	↑↑↑
PCI guidance	↑	↑↑↑	↑↑↑
Radiation dose (mSv)	1–3	N/A	N/A
Spatial resolution (µm)	240 (maximum 110)^162^	100–150	10–15
Cost-effectiveness	↑↑↑	↑	↑↑

Adapted from Nurmohamed *et al*.^163^.

AI/ML, artificial intelligence and machine learning; CCTA, coronary computed tomography; IVUS, intravascular ultrasound; OCT, optical coherence tomography; PCI, percutaneous coronary intervention; TCFA, thin-cap fibroatheroma.

Although ACS caused by plaque erosion differs mechanistically and clinically from plaque rupture,^13^ we continue to manage ACS patients similarly irrespective of the cause. The *EROSION* study highlighted the potential of intracoronary imaging to guide personalized management.^175^ This *proof-of-concept* study enrolled 60 STEMI patients with OCT-detected plaque erosion and <70% coronary stenosis that received anti-thrombotic therapy without stenting. Thrombus volume decreased after 1 month (3.7 vs 0.2 mm^3^), and the vast majority of patients had no ischaemic events during the 4-year follow-up period,^176^ with young patients with little stenosis and thrombosis burden likely deriving the most benefit.^177^ This exploratory study requires validation in a large-scale RCT that would also include NSTEMI powered for outcomes. It nonetheless illustrates the potential of treating a subgroup of patients non-invasively, highlighting the feasibility of a mechanism- rather than ECG-based risk stratification strategy to pave the way for a more personalized ACS management. A point-of-care differentiation of ACS due to rupture vs erosion to determine the need for PCI, for example, by the use of AI/ML analyses of ECG and biomarker data, would tremendously facilitate implementation of such a personalized strategy.

### Soluble biomarkers

Troponin-T and I rise rapidly following myocardial injury, and troponin concentrations remain the reference standard for a diagnosis of acute MI in routine clinical care.^31,32^ The availability of hs-cTn assays and the introduction of rapid *rule-in* and *rule-out* protocols (with a negative predictive value exceeding 99%) now permit prompt diagnosis of MI with high accuracy.^31,32,178^ While hs-cTn levels reflect the extent of myocardial injury and thus risk of death,^179^ troponins originate from the cytosol of injured cardiomyocytes and thus do not reflect any of the mechanisms that actually trigger the ACS.

Our growing understanding of plaque evolution and progression has spurred innovative studies on novel biomarkers with the collective goal to link the mechanistic basis of ACS to future patient management (*Table 4*).^6,180–185,187^ For instance, cathepsin S links experimentally with ECM breakdown and plaque rupture,^188^ and its circulating levels can improve early risk stratification in patients with NSTE-ACS.^180^ Similarly, elevated concentrations of the soluble form of the lectin-like oxidized LDL receptor-1, implicated experimentally in plaque evolution and disrupture,^189^ are associated with increased risk of mortality and plaque progression of non-culprit lesions, as assessed by IVUS.^181^ By harnessing OCT, Yamamoto *et al*.^6^ identified haemoglobin as an independent marker of plaque erosion among 1241 ACS patients, with levels exceeding 15.0 g/dL associating with a 1.48-fold increased risk of plaque erosion as the ACS causing mechanism. While the reason for this relationship remains obscure, haemoconcentration might facilitate endothelial desquamation. Other candidate biomarkers of plaque erosion include MPO and HYAL2-enriched monocytes,^68,190^ and epidermal growth factor and thrombospondin 1, identified by proteomics.^191^ The *OPTICO–ACS study*^69^ ushered in a new era of translational studies to disentangle the mechanistic basis of ACS, providing insights into the role of *TLR2*-mediated neutrophil activation in OCT-detected plaque erosion, which may lend itself for the study of novel diagnostic and eventually therapeutic targets.

**Table 4:** Examples of studies on biomarkers implicated in acute coronary syndrome pathobiology and their relation to clinical outcomes

Biomarker	Pathophysiological context	Type of ACS	Patient numbers	Clinical outcomes	Risk estimates/performance metrics (95% CI)	Reference
Cathepsin S	Implicated in ECM breakdown and plaque rupture	NSTE-ACS	1112	8-Year all-cause mortality 8-Year CV mortality	aHR for upper vs lowest quartile, 1.89 (1.34–2.66) aHR, 2.58 (1.15–5.77)	^180^
Soluble lectin-like oxidized LDL receptor-1	Implicated in plaque progression and rupture	STEMI (53.7%) and NSTE-ACS (46.3%)	2639	1-Year and 30-day all-cause mortality 1-Year and 30-day CV mortality Plaque progression over 1 year (IVUS-assessed)	aHR for upper vs lowest tertile, 3.11 (1.44–10.61) and 2.04 (1.19–3.92) aHR, 3.81 (1.62–19.62) and 2.29 (1.19–5.34) AUC, 0.74 (0.59–0.86)	^181^
Myeloperoxidase	Leukocyte-derived pro-oxidative enzyme linked to NETosis, plaque erosion, and atherosclerosis progression	Not specified	1090	30-Day and 6-month death or non-fatal MI (composite)	aHR, 1.8 (1.1–3.3) and 2.1 (1.7–5.2)	^182^
Interleukin-1β	Downstream of the NLRP3 inflammasome; elicits a variety of pro-inflammatory functions implicated in atherogenesis (e.g. SMC hyperreactivity)	STEMI	1398	90-Day and 1-year all-cause mortality 90-Day and 1-year CV mortality 90-Day and 1-year MACE	aHR for upper vs lowest quartile 2.78 (1.61–4.79) and 1.93 (1.21–3.06) aHR, 2.42 (1.36–4.28) and 2.32 (1.36–3.97) aHR, 2.29 (1.31–4.01) and 2.35 (1.39–3.96)	^183^
Interleukin-6	Likely causally involved in atherosclerosis progression	STEMI (45.4%) and NSTE-ACS (54.6%)	4939	2.5-Year MACE 2.5-Year CV death or heart failure (composite)	aHR per SD increase, 1.10 (1.01–1.19) aHR, 1.22 (1.11–1.34)	^184^
Haemoglobin	Unknown; possibly implicated in shear stress-mediated endothelial denudation/plaque erosion	STEMI (52.2%) and NSTE-ACS (47.8%)	1241	OCT-determined plaque erosion during the index procedure	aOR for >15.0 vs ≤ 15.0 g/dL, 1.48 (1.09–2.01)	^6^
Growth differentiation factor-15	Member of the transforming growth factor beta superfamily (as is growth differentiation factor-11^185^); up-regulated in response to hypoxic, mechanical, oxidative, or inflammatory stress	NSTE-ACS	1122	6-Month all-cause mortality Non-fatal MI Improvement in the discrimination of the GRACE score	HR per SD increment in the natural log scale, 2.4 (1.9–3.0) HR 1.8 (1.2–2.6) Increase in AUC from 0.79 (0.71–0.85) to 0.85 (0.77–0.93)	^186^

Key considerations for the interpretation of biomarker studies include analytical validity, clinical utility, reproducibility, and added value over existing risk models.

ACS, acute coronary syndrome; aHR multivariable-adjusted hazard ratio; aOR multivariable-adjusted odds ratio; AUC, area under the receiver operating characteristic curve; CI, confidence interval; CV, cardiovascular; CK-MB, creatine kinase-MB; ECM, extracellular matrix; GRACE, Global Registry of Acute Coronary Events; HR, hazard ratio; MACE, major adverse cardiovascular events; MI, myocardial infarction; NSTE-ACS, non-ST-segment elevation ACS; OCT, optical coherence tomography; SD, standard deviation; STEMI, ST-segment elevation myocardial infarction.

The identification of a blood-based biomarker reflecting ACS pathobiology is within reach; however, well-designed, large-scale prospective cohort studies combining intracoronary imaging and high-throughput technologies will be required to screen for novel candidates. The adherence to established guidelines for the standardized reporting of observational studies, including diagnostic and prognostic work, can help to identify and mitigate bias, improve transparency, reduce variability, and enhance external validity.^192–194^

### Towards a more pathophysiologically informed management of acute coronary syndromes

While STEMI patients proceed immediately to revascularization, risk stratification for those with NSTE-ACS remains challenging: importantly, up to one-quarter of NSTEMI patients have coronary occlusion responsive to immediate revascularization therapy.^39,195^

Dedicated ACS guidelines endorsed by the *European Society of Cardiology* suggest guiding the timing (immediate vs early [<24 h] vs inpatient vs selective) of an invasive strategy in patients with NSTE-ACS based on classifications of risk as ‘*very high*’ (i.e. haemodynamic instability or CS, refractory chest pain, acute heart failure due to ACS, life-threatening arrhythmias or in-hospital cardiac arrest, mechanical MI-induced complications, dynamic ischaemic ECG changes), ‘*high*’ (i.e. confirmed MI, GRACE >140 points/>3% in-hospital mortality risk, transient ST-segment elevation, dynamic ST-segment/T-wave changes), or ‘*non-high*’ (absence of any risk features with low suspicion of UA).

This scheme does not fully reflect our current understanding of ACS pathobiology. While ST-segment elevation on ECG indicates transmural infarction and thus coronary occlusion, ∼5% of patients with STEMI have no occlusion, while up to ∼25% of NSTEMI patients have occlusive MI.^39,195,196^ Specifically, occlusion of the left circumflex artery or large diagonal branches may result in subtle ECG changes (e.g. ST-depression), often hidden to the human eye.^39^ These findings indicate the need for innovation in the triage of patients with ACS.

The benefit of using the GRACE risk score for early revascularization emerged from a pre-specified analysis of the TIMACS trial^197^ with a subsequent meta-analysis^198^ providing a strong rationale for both the cluster-randomized controlled AGRIS^199^ and UKGRIS^200^ trials. In the prematurely terminated randomized AGRIS trial, the GRACE risk score-based management did not change the 1-year composite endpoint of MI or all-cause mortality.^199^ This result aligns with the large-scale UKGRIS trial in which the implementation of GRACE score-based management did not improve guideline adherence or reduce 1-year MACE in patients with suspected NSTE-ACS.^200^ While these results challenge current guideline recommendations, certain study limitations (e.g. use of the GRACE 2.0 score for the prediction of 6-month MI/mortality with limited performance^23,179,201^; limited power due to early study cessation or lower than expected cluster size) warrant consideration.

### The promise of artificial intelligence-guided tools

Artificial intelligence and machine learning promise to transform many aspects of cardiovascular care, and ACS management in particular, and may also inform primary and secondary prevention measures.^202–204^ Indeed, AI/ML opens novel avenues to link ACS pathobiology to revised management concepts, aid in ECG and imaging data interpretation, guide interventional procedures, and provide accurate outcome predictions with the collective goal to personalize ACS management and improve individual outcomes (*Table 5*).

**Table 5:** Selected artificial intelligence and machine learning-assisted tools to enhance contemporary acute coronary syndrome management

Tool name	Data points	Type of ACS included	Development cohort (*n*)	Validation cohort (*n*)	Outcome predicted	Reference
CoDE-ACS score	hs-cTn, age, sex, pre-hospital delay, chest pain, ischaemic heart disease, hyperlipidaemia, HR, sBP, Killip class, ischaemic ECG, eGFR, haemoglobin	NSTE-ACS	10 038	10 286 (7 cohorts)	MI (Types 1, 4b, or 4c) without ST-segment elevation during index hospitalization	^205,206^
MI^3^	hs-cTn-I, age, sex	NSTE-ACS	3013 (2 cohorts)	7998 (7 cohorts)	MI (Type 1)	^207^
SEX-SHOCK score	Post-PCI TIMI-flow^a^, LM lesion^a^, glycaemia, SBP and PP, HR, Killip class, cardiac arrest, age, LVEF, ST-segment elevation, creatinine, CRP	ACS	35 650	17 887 (2 cohorts)	Cardiogenic shock (in-hospital)	^24^
GRACE 3.0 score	Age, HR, SBP, Killip class, creatinine, cardiac arrest, ST-segment deviation, troponin, GRACE 2.0 risk (in-hospital mortality)	NSTE-ACS	386 591	20 727	In-hospital mortality	^23^
PRAISE score	Age, sex, diabetes, hypertension, hyperlipidaemia, PAD, eGFR, previous MI/PCI/CABG/stroke/ bleeding, malignancy, STEMI, haemoglobin, LVEF, treatment with BB, ACEi/ARB, statins, oral anticoagulation, or PPIs, multivessel disease, complete revascularization, vascular access, PCI with DES	ACS	19 826	3444	1-Year post-discharge all-cause death, MI, or major bleeding (defined as BARC Type 3 or 5)	^208^
ECG-SMART algorithm	12-Lead ECG	ACS (excluding those with cardiac arrest, ventricular tachyarrhythmias, pre-hospital STEMI)	4026	3287	OMI (determined by coronary angiography)	^209^
OMI AI model	12-Lead ECG	ACS	10 543	2263	OMI (determined by coronary angiography)	^210^

ACEi, angiotensin-converting enzyme inhibitors; ACS, acute coronary syndrome; AI/ML, artificial intelligence and machine learning; ARB, angiotensin receptor blockers; BB, betablockers; CABG, coronary artery bypass grafting; CRP, C-reactive protein; DES, drug-eluting stent; ECG, electrocardiogram; eGFR, estimated glomerular filtration rate; hs-cTn, high-sensitivity cardiac troponin; HR, heart rate; LM, left main; LVEF, left ventricular ejection fraction; MI, myocardial infarction; PAD, peripheral artery disease; PCI, percutaneous coronary intervention; PP, pulse pressure; PPI, proton pump inhibitors; OMI, occlusion myocardial infarction; SBP, systolic blood pressure; STEMI, ST-elevation myocardial infarction.

^a^Note that SEX-SHOCK_light_ relies on non-procedural variables only.

Over the last decades, we have moved from a ‘*lumen*-’ to a *‘lesion*’-centred approach,^211,212^ and high-resolution imaging coupled with AI/ML now paves the way for the simultaneous assessment of quantitative and qualitative coronary plaque characteristics to treat the plaque rather than a single stenosis. For instance, Park *et al*.^213^ developed a deep learning (DL) model to suspect plaque erosion on sequential intracoronary OCT images in 581 ACS patients, with subsequent external validation in 292 patients, yielding superior discriminative performance compared with a reference model [area under the receiver operating characteristic curve (AUC), 0.91 vs 0.84, *P* < .001]. By leveraging CCTA data of 395 patients (with the majority having ACS), the same group further developed a DL-based model to identify OCT-detected plaque erosion non-invasively, achieving an AUC of 0.90, with superior performance compared with a reference model (*P* < .05).^168^ Whether such approaches outperform expert clinicians requires further study, but will likely provide a basis for more effective triage of ACS patients in the future.^175–177^

Artificial intelligence and machine learning-based models of ECG analysis provide robust platforms to handle highly dimensional, non-linear ECG data: by harnessing a data set of 7313 US patients presenting with chest pain, Al-Zaiti *et al*.^209^ developed and externally validated a random forest (i.e. an ensemble learning method)-based model for the ECG diagnosis of MI due to coronary artery occlusion, outperforming practising physicians and commercially available ECG interpretation software, which, when combined with clinical expert judgement, correctly reclassified one in three patients. Indeed, the ECG-SMART algorithm improved the detection of occlusion MI, increasing sensitivity by ∼28% and precision by ∼32% points compared with reference methods. Herman *et al*.^210^ developed an DL algorithm on ECGs obtained from 10 543 ACS patients, achieving an AUC of 0.95 and 0.90 in internal and external validation data sets, thereby outperforming conventional STEMI criteria for the diagnosis of occlusive MI.

Artificial intelligence and machine learning-based algorithms for ACS diagnosis may revolutionize early triage decisions. Doudesis *et al*.^205^ have introduced the CoDE-ACS score, an XGBoost (i.e. a decision-tree based model)-based algorithm to predict the probability of MI by integrating hs-cTn levels, age, sex, pre-hospital delay, presence of chest pain, documented ischaemic heart disease, hyperlipidaemia, heart rate, systolic blood pressure, Killip class, ischaemic ECG, renal function, and haemoglobin levels. Trained on 10 038 and validated in 10 286 patients, CoDE-ACS achieved an impressive AUC of 0.95 (with a negative predictive value and sensitivity of 99.7% and 99.0%, respectively), thereby identifying more patients as low risk compared with traditional hs-cTn thresholds, irrespective of timing if measured serially, ruling out more patients than the ESC 0-h pathway.^205,206^ Importantly, however, upon external validation, the negative predictive value of the ESC 0-/1-h algorithm was nominally higher, necessitating a thorough evaluation in independent datasets and potentially further refinements to overcome these safety concerns. That GRACE risk score-based early triage has not provided clinical benefit in contemporary RCTs^199,200^ mandates the need for novel algorithms for the prognostication of adverse events post ACS. By harnessing XGBoost on 386 591 patients with NSTE-ACS, Wenzl and Kraler *et al*.^23^ developed the sex-specific GRACE 3.0 score, achieving an AUC of 0.87 and 0.91 in female and male patients for the prediction of in-hospital mortality during external validation in 20 727 NSTE-ACS patients, thereby classifying more females as high risk (i.e. >3% in-hospital mortality risk), for whom early revascularization is currently recommended.^31,32^ In more recent work, given the nearly unchanged mortality rates of those with ACS complicated by CS,^214^ Yang *et al*.^24^ have introduced the AI/ML-guided and CRP-relying SEX-SHOCK score for the sex-specific risk prediction of in-hospital CS in patients presenting with ACS, thereby allowing accurate risk stratification based on individual CS risk demonstrating improved performance over a reference model.^24^

The advent of AI/ML-assisted tools should improve ACS management considerably, but will require continued research to scrutinize, validate, and further refine newly developed algorithms in well-designed studies to demonstrate rigorously that their use improves long-term outcomes.

## Conclusions: management implications, challenges, and future opportunities

To strive towards a more personalized management in patients with ACS, we must (i) deepen clinical applications of the implications of current mechanistic understanding of ACS, (ii) strengthen our international research efforts (e.g. by building large-scale, multi-national trial consortia and registries) towards a better characterization of patient populations with rising incidence rates and/or plateauing mortality rates (e.g. those with premature ACS, without SMuRFs, or with CS), (iii) conduct innovative trials guided by AI/ML-based tools (e.g. CoDE-ACS, GRACE 3.0, SEX-SHOCK scores), (iv) adopt designs of trials and frame their questions reaching beyond traditional classifications (e.g. STEMI vs NSTE-ACS), and (v) ensure the broad availability and implementation of currently underused technologies, including intracoronary imaging and novel AI/ML-based tools pending independent validation. While existing clinical guidelines do not yet differentiate ACS management based on plaque morphology, precision medicine approaches that are informed by ACS pathobiology rather than a binary ECG/biomarker data interpretation hold immense promise to improve ACS care.

How can we achieve these goals? First, we need to continue our experimental efforts to disentangle the mechanistic basis of increasingly prevalent triggers of ACS, including the eroded plaque. Our experimental focus on the role of inflammation in atherosclerosis has stimulated seminal RCTs.^215–219^ Blockade of IL-1β by canakinumab reduced recurrent events in individuals with chronic coronary artery disease and residual inflammation gauged by C-reactive protein levels.^217^ Discordant to the results of the COLCOT and LoDoCo2 trials, however,^215,216,218^ non-targeted anti-inflammatory strategies with colchicine failed to provide benefits in the large-scale CLEAR-SYNERGY trial.^219^ Although differences in dosing and timing and other considerations may have contributed to the neutral results (reviewed in detail by Bonaventura *et al*.^220^),^221,222^ it is important to consider that most STEMIs are caused by plaque rupture, a macrophage-dominated process, whereas colchicine predominantly affects neutrophil functions.^223^ Given that a NET production dampening effect was observed by colchicine in patients with ACS,^224^ an RCT comparing its efficacy and safety in ACS caused by plaque erosion vs rupture would be an intriguing avenue for future research. In parallel, research efforts on targeted anti-inflammatory strategies should be continued. The ongoing ARTEMIS trial will determine whether targeted IL-6 inhibition improves outcomes in patients with ACS.^225^ Other strategies to interfere with other pathways (e.g. NET formation) also require exploration. In this regard, studying the efficacy and safety of pharmacological MPO inhibition in plaque erosion vs rupture deserves focus.^226,227^ Importantly, any such benefits would need to be incremental to intense LDL-C lowering,^111,112,228,229^ and well-designed RCTs are warranted to determine the optimal revascularization, anti-thrombotic, and lipid-lowering strategies tailored to plaque phenotype.

Second, incident rates of premature ACS have plateaued,^65^ as have mortality rates of those with CS complicating ACS;^213^ thus, we need to redouble efforts to establish well-designed international registries that include previously underrepresented patient populations (also considering sex, race, ethnicity, among others).^17,24,154^ Third, dedicated RCTs on AI/ML-assisted tools are urgently warranted, since many ongoing studies are non-randomized, deviate from reporting standards, and thus have a risk of bias.^230^ Therefore, the code for AI/ML algorithms should be made accessible, to minimize ‘*black box*’ concerns.^203^ Fourth, supported by validated AI/ML-assisted tools,^209,210^ rigorous RCTs evaluating the efficacy and safety of novel paradigms (e.g. occlusive MI/non-occlusive MI vs STEMI/NSTE-ACS paradigm) to triage patients for early revascularization should be performed, since up to one-fourth of NSTEMI patients may have MI with coronary artery occlusion.^39^ Fifth, despite acceptable safety and demonstrated utility,^174^ the clinical adoption of indicated intracoronary imaging has lagged. For example, the *IVUS-ACS* trial^173^ supports its use in the acute setting, informing stent optimization and tailoring of management according to underlying mechanisms.^175,176^ In the future, we might also consider treating non-flow-limiting plaques that harbour characteristics of vulnerability (a frequent substrate for ACS^167^), as shown by the *PREVENT* trial, a notion in need of further validation in large, long-term RCTs with ‘*hard*’ endpoints.^231^

We have entered an exciting era in which novel ‘*disease-modifying*’ pharmacotherapies that directly alter the biological processes within the atheroma have emerged. The integrated use of emerging technologies (e.g. high-resolution CCTA) and AI/ML-assisted tools (e.g. DL-based models for imaging interpretation, ACS diagnosis, or prognostication) may—pending randomized evaluation—transform clinical practice, accelerate the personalized management of the patients with ACS, and provide novel avenues to the improvement in patient outcomes.

## Declarations

### Data Availability

No data were generated or analysed for or in support of this paper.
